# How Can Personalized Medicine Improve Assisted Reproduction Technology Outcomes?

**Published:** 2017

**Authors:** Mohammad Reza Sadeghi

Personalized medicine or precision medicine was first defined for management of cancer patients with the idea that information of patient’s genes, proteins, metabolites and environment can be applied for its individualized management and it encompasses prevention, screening, diagnosis, prognosis, treatment, follow up, detection of recurrence and categorization of patient to defined subgroups for more effective treatment. Current evidence suggests the relative success of this approach in cancer patients. The development and implementation of personalized medicine required a set of objectives and valid biomarkers through genomics, transcriptomics, proteomics and metabolomics knowledge of huge samples cohorts regarding confounding factors, such as age, gender, habits, diet, and environment. For the first time, human genome sequencing cost up to $3 billion; however, it costs less than $5000 recently and it continues to decline in price and time rapidly. This is the direct approach for genetic biomarker discovery through genome and transcriptome but the current metabolomics and proteomics techniques require high expertise, labor, and infrastructure, and therefore are more expensive than genomics and transcriptomics technologies at present (1).

Personalized medicine in reproductive medicine is still in its nonage since it is not yet a fully strengthened mature arena. Reproductive medicine is at the priliminary stage of discovering and validating genomic, protein and metabolite biomarkers. Perhaps what is currently being offered as personalized treatment of infertile patient is more based on “the best expert opinion of the attending clinician” than “the best evidence-based data available”. Clinicians from all disciplines now accepted the concept “one size does not fit all” accordingly to their practice in diagnosis and treatments should consider patients’ specific molecular profiles. The past and present “one size fits all” practice would inescapably hamper some patients from more efficient treatments. Albert Einstein wrote: “If you want different results, do not do the same”. This concept is representative of our current practice in reproductive medicine; “failed IVF cycles followed by repeating the same cycles for all couples”. Thus, the consequence of this approach is facing with hordes of infertile couples as the victims of repeated implantation failure (RIF) (2).

Historical facts show that inability of reproductive medicine in applying personalized therapy for efficient treatment is remunerated by transfer of numerous embryos to guarantee higher chance of successful pregnancy. The consequence of this practice is dangerous situations of multiple pregnancies in %30–40 of IVF cycles (3). At the first glance, infertility treatment suffers from lack of standardization in the field reproductive medicine. The most evident variations are in oocyte and embryo grading, endometrial assessment, semen analysis, and also in diagnosis and treatment within and between clinics, technicians and physicians that lead to varying success rates between clinics and even different physicians in one clinic. Another feature of reproductive medicine is that it is not “personalized” to only one individual but in reality, you are faced with at least three different individuals; the mother, father and embryos. Another complication is personalization of different biological systems; the egg, sperm, embryo and uterus. Even more importantly, one or more systems may be involved in etiology of infertility and its efficient treatment in a particular couple with great impact on treatment outcomes (3).

Preliminary data on large cohorts of samples revealed genetic biomarkers linked to spontaneous ovulation for PCOS patients, efficacy of low molecular weight heparin for the prevention of spontaneouse abortion, specific allells of FSH receptor linked with OHSS or poor response to controlled ovarian hyperstimulation. The other clinically available biomarkers are anti-Mullerian hormone (AMH) levels and antral follicle count (AFC) for tailoring gonadotropin dosage, and also the expression of 238 genes as the transcriptomic signature of endometrium entitled as endometrial receptivity array (ERA). It personalized embryo transfer via personalization of implantation window and determination of proper time for embryo transfer in patients with repeated implantation failures (3, 4).

Finally, reproductive medicine requires identification and verification of ideal biomarkers that personalize the entire process to make possible convenient preventive and/or preferable targeted therapy including prevention, screening, diagnosis, prognosis and treatment such as prediction of ovarian reserve, stimulation outcome, gametes and embryos quality, endometrial receptivity, ectopic pregnancy, and probability of pre-eclampsia and preterm labor. Therefore, the answer to above question would not be very accurate in the present situation. It requires identification of new biomarkers from genomics, transcriptomics, proteomics, and metabolomics data in several available large databases and subsequently verification and validation of each biomarker through prospective large randomized clinical trials.
